# Release of Ammunition-Related Compounds from a Dutch Marine Dump Site

**DOI:** 10.3390/toxics11030238

**Published:** 2023-03-01

**Authors:** J. H. den Otter, D. Pröfrock, T. H. Bünning, J. S. Strehse, A. E. D. M. van der Heijden, E. Maser

**Affiliations:** 1 Department of Energetic Materials, TNO, Ypenburgse Boslaan 2, 2496 ZA The Hague, The Netherlands; 2 Department Inorganic Environmental Chemistry, Institute of Coastal Environmental Chemistry, Helmholtz-Zentrum Hereon, Max-Planck-Straße 1, 21502 Geesthacht, Germany; 3 Institute of Toxicology and Pharmacology for Natural Scientists, University Medical School Schleswig-Holstein, Brunswiker Straße 10, 24105 Kiel, Germany

**Keywords:** sea dumped munitions, environmental damage, toxicity, energetic compounds, contamination by metals, Eastern Scheldt

## Abstract

After World War II, large amounts of ammunition were dumped in surface waters worldwide, potentially releasing harmful and toxic compounds to the environment. To study their degradation, ammunition items dumped in the Eastern Scheldt in The Netherlands were surfaced. Severe damage due to corrosion and leak paths through the casings were observed, making the explosives in the ammunition accessible to sea water. Using novel techniques, the concentrations of ammunition-related compounds in the surrounding seabed and in the seawater were analyzed at 15 different locations. In the direct vicinity of ammunition, elevated concentrations of ammunition-related compounds (both metals and organic substances) were found. Concentrations of energetic compounds ranged from below the limit of detection (LoD) up to the low two-digit ng/L range in water samples, and from below the LoD up to the one-digit ng/g dry weight range in sediment samples. Concentrations of metals were found up to the low microgram/L range in water and up the low ng/g dry weight in sediment. However, even though the water and sediment samples were collected as close to the ammunition items as possible, the concentrations of these compounds were low and, as far as available, no quality standards or limits were exceeded. The presence of fouling, the low solubility of the energetic compounds, and dilution by the high local water current were concluded to be the main causes for the absence of high concentrations of ammunition-related compounds. As a conclusion, these new analytical methods should be applied to continuously monitor the Eastern Scheldt munitions dump site.

## 1. Introduction

In the decades after World War II, large amounts of leftover ammunition were dumped in surface waters around the world. In the Netherlands, the main dump sites are located in the North Sea near Hook of Holland and IJmuiden, in the Eastern Scheldt (in Dutch: Oosterschelde) near Zierikzee, and in the Wadden Sea between Ameland and Schiermonnikoog. Due to the components present in ammunition, such as warfare agents, explosives, and heavy metals, sea-dumped ammunition is a potential source of pollution of the environment. The nearby fishery and mussel farming, as close as 200 m north of the dump site and 1000 m west, makes monitoring of the Eastern Scheldt dump site crucial.

For the last two decades the Netherlands’ Organization for Applied Scientific Research (TNO) was involved in monitoring the ammunition dump site in the Eastern Scheldt [1,2,3]. In 1999, the ammunition on the seabed was studied using sonar and an underwater camera, a few ammunition items were surfaced, and water and sediment samples were collected in and close to the dump site [4]. The ammunition items were localized and surfaced by divers and were found partly buried in the seabed. However, the local tidal currents may have caused the extent of coverage by the seabed to vary over the past decades. Ammunition casings were found to be affected by the sea water, and elevated concentrations of ammunition-related compounds like 2,4-dinitrotoluene, diphenylamine, copper, zinc, and lead were detected at several locations. In 2000, a desk study based on historical documents was performed, aiming to estimate the size and contents of the dump site [5]. The size was concluded to be 30,000 metric tons and a wide range of ammunition types was found to be dumped, including mines, aircraft bombs, bullets, and smoke grenades. Based on the degradation of ammunition recovered from a dump site in the North Sea, the time required for the release of the total amount of the explosive substances in this dump site was estimated to be 500 years [6]. In 2001, water and sediment samples were collected again in the Eastern Scheldt dump site and analyzed using more advanced techniques, again showing the presence of traces of explosives. Active biomonitoring using mussels (*Mytilus edulis*) was also applied by analyzing the accumulated amounts of ammunition-related compounds after 6 weeks exposure in the ammunition dump site. The detected amounts were concluded to be insufficient to pose human health issues, even after daily consumption of these mussels [7]. In 2003, sediment samples were collected for the determination of ammunition-related compounds and for studying the number and species of marine life [8]. No correlation was found between the presence of ammunition-related compounds and deviations in the species composition.

In 2014, water samples were collected by Rijkswaterstaat, the organization responsible for water management in the Eastern Scheldt. Samples were collected at 1 m above the seabed, halfway down the water column, and at 1 m below sea level [9]. For lead and iron, higher concentrations were observed at 1 m above the seabed than higher in the water column. The water samples were analyzed for nitrotoluenes and nitro-benzenes. These compounds were not detected; however, no detection limits were reported. The study concluded that no water quality limits were found to be exceeded above the dump site; however, information on which water quality limits were used for comparison was not given.

In 2014, the concentrations of heavy metals and explosives in Japanese oysters were studied by IMARES [10]. These oysters were collected at the eastern edge of the Eastern Scheldt dump site and their tissue was analyzed for explosives with detection limits of 5 ng/g wet tissue for 2,4,6-TNT and its metabolites and 10 ng/g tissue for RDX and HMX. No explosives were detected in the oysters and the concentrations of heavy metals were, except for copper, not higher than in oysters collected at reference locations. No risk levels for ecology and human food safety were exceeded by the detected concentrations of ammunition-related compounds.

The present study is part of a continuous monitoring of the Eastern Scheldt munitions dump site and was conducted because, after over 50 years, the thin-walled casings of ammunition shells like anti-tank mines and warheads were found to have partially disappeared, or for ammunition items with larger wall-thicknesses, potential leak paths through the metal casings were observed. However, despite these facts, the energetic content consisting of 2,4,6-TNT or mixtures thereof appeared not to be washed out, leading to the question on the contamination status of the surrounding water and sediment. In 2020, a monitoring campaign was executed where ammunition items were surfaced, and water and sediment samples were collected. New methods for analyzing nitroaromatic explosives in water, sediment, and biota were recently developed at Kiel University. With these new methods, important improvements on the limits of detection (LoD) and the limits of quantification (LoQ) have been achieved [11] and have been applied to analyze the samples collected at the Eastern Scheldt. The results of this research were reported to the Dutch Government [12] and are described in this paper.

## 2. Materials and Methods

**Figure 1:**
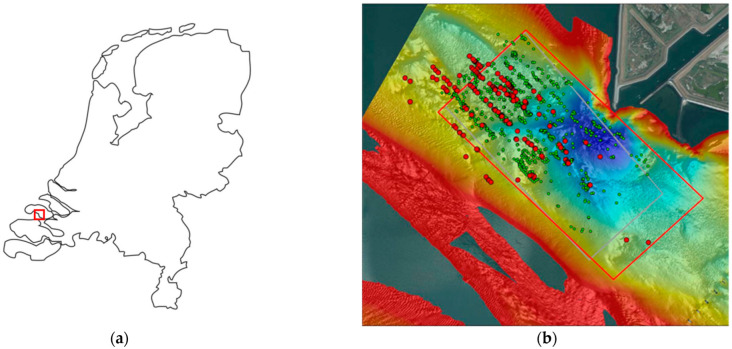
Maps with (**a**) the location of the Eastern Scheldt ammunition dump site in the Netherlands and (**b**) including red dots indicating ammunition visually observed during a ROV survey in 1999 and green dots indicating objects identified using side scan sonar in 1999. Colors indicate depths of −55 m NAP (purple) up to −10 m NAP (red). Reprinted with permission from Ref. [4]. 1999, TNO.

The ammunition dump site studied in this paper is located in the former estuary Eastern Scheldt, about 12 km inland from the North Sea. Due to its open connection to the North Sea, the Eastern Scheldt has tidal currents up to 1 m/s and oxygen-rich salt water (17 mg Cl-/L). The water depth at the dump site is 10 to 55 m and the water temperature at the seabed was 22 °C during the sample collection in August 2020. Due to the combination of the water current; the local water depth; the underwater view, which is limited to 0.5 m; and the ammunition being at least partially buried in the seabed, localizing and surfacing ammunition items is a challenging operation. The location of the ammunition dump site in the Netherlands and relative to the harbor channel of Zierikzee is shown in Figure 1. Figure 1b indicates the ammunition observed during a survey in 1999. 

**Figure 2:**
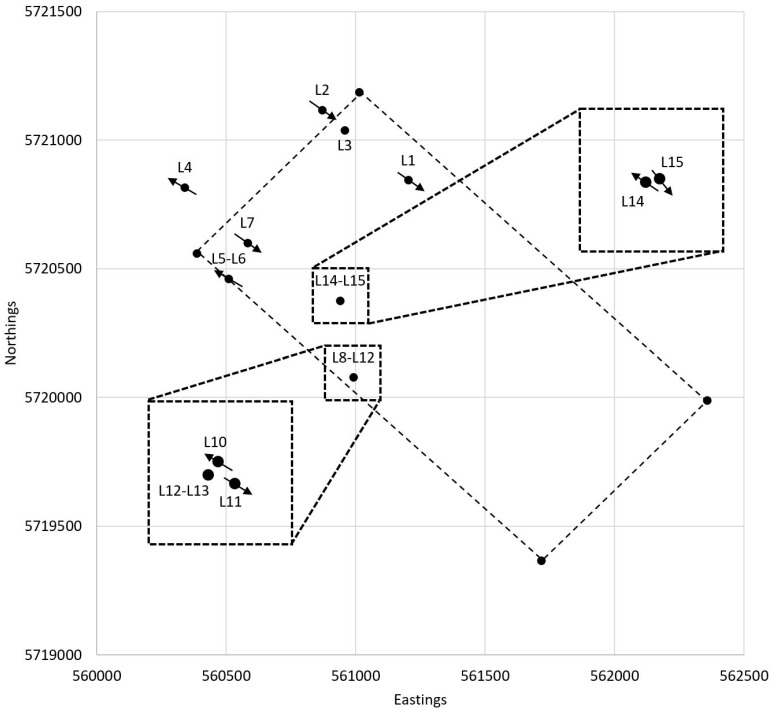
Map of the ammunition dump site in the Eastern Scheldt indicating diving locations (L1–L15) and current direction (arrow) at the moment of diving. No arrow indicates negligible current. Water samples were collected at 20 cm above the seabed. The water sample at location L13 was collected at the surface.

Figure 2 shows the locations where ammunition was surfaced and water and sediment samples were collected in 2020. In total, water samples were collected at 10 locations and sediment samples were collected at 11 locations. Surfacing of ammunition items and water and sediment collection were performed by divers from the Maritime Explosive Ordnance Disposal service. Diving was executed around the turn of the tide to facilitate a workable environment for the divers. After descending, the divers first collected the water samples, then the sediment samples, and after that started searching for and surfacing ammunition.

After surfacing, the ammunition was identified, cleaned, and cleared of explosives. The remaining wall thickness was studied by making cross-sections of the ammunition casings using a diamond wire saw.

**Figure 3:**
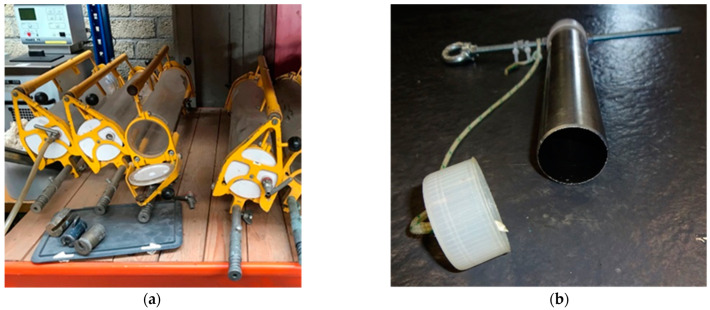
Tools for water and sediment sample collection: (**a**) water sample collection bottles with a volume of 3 L, and (**b**) tubes for sediment sample collection with a diameter of 40 mm. Samples for metal analysis were collected using polypropylene tubes.

Water samples were collected using water-sample collection bottles, shown in Figure 3. The bottle was taken to the seabed by a diver with both caps open. At 20 cm above the seabed, the bottle was allowed to flush with sea water, and subsequently, the bottle was closed. For reference purposes, water samples were also collected at the water surface above the dump site and 20 km inland from the dump site. Immediately after surfacing the bottle, the sample was divided in two aliquots. For analysis of organic compounds, a 1 L aliquot was, after addition of 200 µL of an isotopically labeled 250 µg/L 2,4,6-TNT in acetonitrile solution, extracted over a Chromabond Easy solid phase column at 4 °C. After extraction, the columns were cooled to −18 °C and protected from light until the day of analysis. For metal analysis, a 0.5 L aliquot was, directly after surfacing, transferred to a pre-cleaned polypropylene bottle, cooled to −18 °C, and protected from light until the day of analysis.

Sediment samples were collected close to ammunition items observed in the seabed by a diver pushing a cleaned sampling tube approximately 20 cm deep into the seabed. Immediately after retraction from the seabed, the tube was closed using a cap and surfaced. The content of the tube was homogenized, transferred to a plastic bag, and cooled to −18 °C until the day of analysis. For the collection of samples for metal analysis, cleaned polypropylene tubes were used to prevent contamination of the samples.

### 2.1. Sample Preparation for Energetic Compounds Analysis

SPE (solid phase extraction) columns of water samples were thawed in the laboratory, dried in a light vacuum for 30 min, and eluted with 3 mL acetonitrile (ACN). The eluates were pre-concentrated to 600 µL and measured by GC-MS/MS for energetic compounds and diphenylamine (DPA). For the analysis of hexachloroethane (HCE), 20 µL of the solution was diluted with 80 µL of methylene chloride and measured separately. 

Twenty grams of sediment were lyophilized for 18 h. Two times 2 g of each sediment were weighed into centrifuge tubes for the energetic compounds/diphenylamine and hexachloroethane analyses, mixed with 5 mL of ACN (explosives and DPA analyses) or methylene chloride (HCE analyses), mixed for 1 min using a vortex mixer, sonicated for 15 min, and centrifuged for 10 min (4100 rpm at 10 °C). Supernatants were filtered through 0.2 µm PTFE filters and concentrated to 600 µL.

All samples were stored at −20 °C until measurement. Organic compounds were measured on a Thermo Scientific TSQ 8000 Evo triple quadrupole tandem mass spectrometer in selected reaction monitoring (SRM) mode, coupled to a TRACE1310 gas chromatograph equipped with a TG-5MS column (30 m × 0.25 mm × 0.25 μm). The measurements were carried out using a split/splitless liner in splitless mode and a quartz wool liner. The GC-MS/MS parameters used for both methods are given in Table 1. 

Sample concentrations of the explosives were determined by external calibration using standard dilutions of 0.1–100 ng/mL in ACN, 0.1–25 ng/mL in ACN for DPA, and 0.01–10 ng/mL in DCM for HCE. Analyte loss during extraction was further corrected using the internal ^13^C^15^N-TNT standard. For the water samples, 50 ng/L ^13^C^15^N-TNT was used as an internal standard. The GC retention times and MS/MS transitions used for quantification are listed in Table 2. Recording and interpretation of the spectra was done in TraceFinder 4.1.

### 2.2. Sample Preparation for White Phosphorus Analysis

Five grams of wet sediment were transferred into 20 mL screw top headspace vials with 10 mL Millipore water. Extraction was carried out with a gray PDMS (polydimethylsiloxane) fiber, using a TriPlus RSH autosampler with an automated solid phase microextraction (SPME) probe head. The samples were incubated for 30 min at 60 °C and extracted for 10 min. A Thermo Scientific TSQ Duo triple quadrupole MS coupled to a TRACE 1310 with a splitless injector with an SPME liner was used. The injector was kept at a constant temperature of 290 °C. Splitless time was set to 1 min. After injection, the fiber was baked out for 5 min in the injector. For quantification, the P4 peak was recorded in single ion monitoring mode, with the P3 fragment in secondary reaction monitoring as the confirming peak. A freshly prepared standard dilution of P4 (0.05–5 ng/mL in Millipore water) was used for quantification. Spectra were recorded and analyzed using Chromeleon 7.2. GC-MS parameters are given in Table 1, retention times and quantification peaks are shown in Table 2.

### 2.3. Sample Preparation for Trace Metal Analysis and Instrumental Setup

Preparatory laboratory work was performed in a class 10,000 clean room inside a class 100 clean bench. 

The sea water samples were pressure filtrated in triplicates inside a class 100 clean bench using pre weighted 0.45 µm Whatman Nucleopore track etched polycarbonate membranes. After filtration, the samples were acidified with doubly subboiled concentrated nitric acid and stored in acid cleaned LDPE bottles until further analysis. Multi-elemental analysis of the sea water samples was performed using an Agilent 8900 inductively coupled plasma tandem mass spectrometry (ICP-MS/MS) coupled on-line with a fully automated matrix removal and preconcentration system for seawater analysis (ESI seaFAST SP2). Briefly, the sea*FAST* was equipped with two chelating columns filled with Nobias chelate-PA1 (HITACHI High-Tech Fielding Corporation, Ibaraki, Japan) resin, allowing the removal of the interfering salt matrix of the sample as well as its preconcentration to improve detection limits for low abundance analytes. The calibration solutions were automatically diluted from two multi-element solutions (1 ng L^−1^ and 1000 ng L^−1^) via the syringe module of the sea*FAST* SP2 to minimize possible contaminations and dilution errors of the standards during preparation. For further information on the sea*FAST* SP2 see Ebeling et al. [13]. The ICP-MS/MS was equipped with s-lenses and operated in single quadrupole mode with He/H_2_ (4 mL min^−1^ and 0.5 mL min^−1^, respectively) as cell gases to further minimize interferences. To validate the method, certified seawater reference material (NASS-7; NRC, Ottawa, Ontario, Canada) was measured on a regular basis with the samples. Sample concentrations of the dissolved metals were determined by external calibration using the standard dilution series of NIST traceable multi element solutions ranging from 1 to 1000 ng/L in 1% subboiled HNO_3_. Ir and Rh were used as internal standards to account for instrumental drift during long measuring series.

Multielemental data were processed using MassHunter version 4.2.

The homogenized sediment samples were freeze-dried and milled to obtain a homogeneous fine powder. Each homogenized sample was digested in triplicates by digesting about 50 mg of the sample with 5 mL nitric acid, 2 mL hydrochloric acid, and 1 mL fluoroboric acid for 300 min at 180 °C with a MARS 5 Xpress microwave. After digestion, the solutions were transferred quantitatively to 50 mL pre-cleaned DigiTUBEs and diluted to a final volume of 50 mL with Milli-Q water. Elemental concentration determination in the sediment digests was performed using an Agilent 8800 inductively coupled plasma tandem mass spectrometer (ICP-MS/MS) coupled to an ESI SC-4 DX FAST autosampler. H_2_, He, O_2_, and NH_4_ were used as cell gases for the octopole reaction cell of the ICP-MS/MS to minimize potential interference due to the sediment matrix, which influences the accurate determination of the selected trace metals and metalloids. Mass fractions of the metals in the sediments were determined by external calibration using the standard dilution series of NIST traceable multi element solutions ranging from 0.01 to 100 µg/kg and 0.1 to 1000 µg/kg, respectively, in 1% subboiled HNO_3_. Ir and Rh were used as internal standards to account for instrumental drift during long measuring series. Further information on the digestion and analysis method can be found at [14]. 

## 3. Results

### 3.1. Ammunition Items Recovered

**Figure 4:**
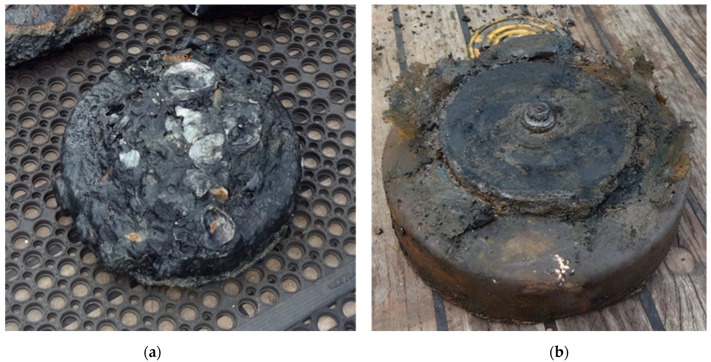
Images of a surfaced anti-tank mine: (**a**) as found on the seabed and (**b**) after removal of fouling.

A wide range of ammunition types was surfaced, including anti-tank mines, high-explosive and smoke grenades, bullets, warheads, and propellant cartridges. Most ammunition items were at least partially covered in the seabed and were present in packaging like wooden or metal ammunition boxes. Ammunition items were generally covered by fouling as shown for an anti-tank mine in Figure 4. 

**Figure 5:**
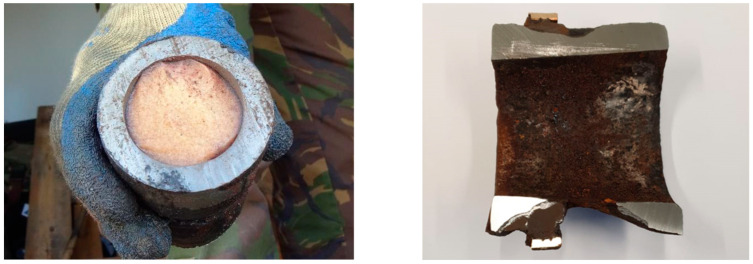
Cross-section of a high-explosive 75 mm recoilless rifle grenade found on the seabed, before and after removal of the energetic content.

After removal of the fouling, thin metal casings, like for a mine or a warhead, were found to be largely affected or had largely disappeared. In addition, significant damage was observed due to galvanic corrosion at the location of screw threads or a driving band, as shown for a grenade casing with an original wall thickness of 13 mm in Figure 5. 

Although the explosive content was no longer enclosed by the metal casing, the content appeared to be intact, as shown in Figure 4 for an anti-tank mine and in Figure 5 for a grenade.

### 3.2. Organic Substances

The energetic content of high-explosive ammunition items used during World War II was mainly 2,4,6-trinitrotoluene (2,4,6-TNT) and mixtures thereof. A widely used stabilizer used in gun propellants is diphenylamine (DPA). In smoke ammunition, mainly hexachloroethane (HCE) and white phosphorus (WP) were used. All of these compounds and some of their degradation products could have an impact on the marine environment. The water and sediment samples collected during this study were analyzed for the presence of 2,4,6-TNT, its decomposition products 2,4-dinitrotoluene (2,4-DNT), 2-amino-4,6-dinitrotoluene (2-ADNT), 4-amino-2,6-dinitrotoluene (4-ADNT), and 1,3-dinitrobenzene (1,3-DNB), for DPA, and for HCE. The analysis for WP is described in Section 3.4.

#### 3.2.1. Water Samples

The amounts of organic substances detected in the water samples, as well as the detection and quantification limits, are shown in Table 3. 

While in 2020 the analytical determination had significantly been improved, resulting in LoD and LoQ values in the one-digit nanogram per liter range and below, detection and quantification limits were in the low microgram per liter range in the year 1999 and around 10 nanograms per liter in 2001. For 2,4,6-TNT, a minimum concentration of 3 ng/L could be observed in the blank samples, causing the higher detection limit for this substance. The relatively high detection limit for DPA was caused by the release of small amounts of this substance from the extraction columns. In 1999 and 2001, DPA was not analyzed at all. Hence, in the present report we are able to give relatively detailed data on the concentrations of nitroaromatic explosives occurring in the water body of our study area.

In all water samples from year 2020, 2,4,6-TNT was the most abundant energetic compound (56.7 ng/L at location L7; 44.5 ng/L at L6; 29.1 ng/L at L12), while the 2,4,6-TNT metabolites 2,4-DNT, 2-ADNT, 4-ADNT, and 1,3-DNB and HCE were comparatively less abundant. As expected, at locations with increased 2,4,6-TNT concentrations, increased concentrations of its transformation products 2-ADNT and 4-ADNT were also observed. Here, TNT concentrations were generally 4- to 7-fold higher than its transformation products 2-ADNT and 4-ADNT (Table 3). DPA was only detected at location L14.

#### 3.2.2. Sediment Samples

The amounts of organic substances found in the sediment samples, as well as the detection and quantification limits, are shown in Table 4. 

As was described for the water samples, the methodology for the analytics of nitroaromatics had significantly been improved in the year 2020. However, due to the variant pretreatment method, detection limits for sediment samples are higher than for water samples; note that values for the sediment concentrations of nitroaromatics are given as per gram, resulting in LoD and LoQ values below the one-digit nanogram per gram range.

In most sediment samples, the concentrations of 2,4,6-TNT and its transformation products were below the detection or quantification limit. Only at locations L6 and L11 were both 2,4,6-TNT and transformation products thereof found, indicating the release of 2,4,6-TNT in the vicinity of these locations. In most of the sediment samples, both DPA and HCE were detected, up to concentrations of 7 ng/g and 0.7 pg/g, respectively. 

### 3.3. Metals

Metals are present in both ammunition casings and in some of the energetic compounds used in detonators, like lead azide. High concentrations of heavy metals released from degrading or leaking ammunition items could affect the marine environment. The concentrations of iron (Fe), copper (Cu), nickel (Ni), chromium (Cr), zinc (Zn), tin (Sn), molybdenum (Mo), cobalt (Co), lead (Pb), and cadmium (Cd) were analyzed in the water and sediment samples. 

#### 3.3.1. Water Samples

The metal concentrations detected in the water samples are shown in Table 5. 

Due to the method applied for removing the sea water matrix, Cr could not be analyzed. The observed metal concentrations were similar for most locations. At some locations, concentrations up to a factor 10 higher were observed, like Sn at L1, Cd at L4, Pb at L11 and L14, and Zn at L4, L11, and L14. Cu and Zn concentrations were significantly higher in the samples collected at 20 cm above the seabed than at L13, where the sample was collected at the surface. The increased concentrations of several metals at L4, L10, and L11 suggest that release of metals occurs at these locations, potentially from leaking ammunition items. 

#### 3.3.2. Sediment Samples

The amounts of metals found in the sediment samples are shown in Table 6. 

For most of the sediment samples, the metal concentrations were similar, except for the Zn concentrations at L6 and L7 and the Cu concentrations at locations L10, L14, and L15. 

### 3.4. White Phosphorus

The sediment samples collected during this study were also analyzed for the presence of white phosphorus. The detection limit for the analysis method used was 0.03 nanograms per gram of dry sample. In none of the analyzed sediment samples white phosphorus was detected. Additionally, during the monitoring studies at this dump site in 2002 and 2004, with similar detection limits, no white phosphorus was detected in the sediment samples.

## 4. Discussion

### 4.1. The Eastern Scheldt Dump Site

Overall, the ammunition items detected during this research were found to be significantly affected. After over 50 years in the Eastern Scheldt, the thin-walled casings of ammunition shells like anti-tank mines and warheads were found to have partially disappeared. In addition, for ammunition items with larger wall-thicknesses, potential leak paths through the metal casings were observed at the location of driving bands and the screw threads due to galvanic corrosion. Although the casings did not fully enclose the energetic content anymore, the energetic content consisting of 2,4,6-TNT or mixtures thereof appeared to not be washed out. The presence of fouling, the only partially permeable casings, and the low solubility of the energetic compounds may be an explanation for the energetic content to be found mostly intact despite the presence of a leak path through the casing. 

Despite the leak paths present, only low concentrations of ammunition-related compounds, like metals, energetic compounds, and their transformation products, were found in the surrounding water and sediment samples collected in the ammunition dump site, even though water samples were collected at only 20 cm above the seabed and sediment samples were collected close to ammunition items observed in the seabed. Due to the open connection to the North Sea, tidal current is present at the dump site in the Eastern Scheldt. Because of this, released ammunition-related compounds will be diluted, and in the oxygen-rich water of about 20 °C compounds like 2,4,6-TNT will probably be transformed to metabolic intermediates. It is known that the environmental fate of energetic compounds is determined by physio-chemical properties such as their solubility and octanol-water partitioning coefficients, or by the temperature, pH, salinity, oxygen dissolution, UV light levels, and the ionic strength of the seawater [15,16]. The dissolution fluxes determined in a study by Beck et al. [17] show large variability, ranging from 1 to 3 mg × cm^−2^ × day^−1^. They conclude that the surface of exposed underwater munitions will retreat at a low rate on the order of 1–5 mm per year. In addition, degradation by microbial communities are also factors that influence the occurrence of munitions compounds at a dump site [18]. 

The amount of ammunition dumped in this area was estimated to be 30,000 metric tons; however, based on a survey executed in 1999 [4], less than 1% of the ammunition is expected to be lying on top of the seabed. The majority of the ammunition is expected to be partially or even completely buried in the seabed, and, due to the lower oxygen concentrations, degradation of ammunition casings is expected to be slower there. 

Although increased concentrations of ammunition-related compounds like heavy metals and explosives were found at several locations, no clear correlations could be found between the presence of ammunition at these locations and the increased concentrations. For some locations, like L4, L6, L10, L11, and L14, increased concentrations of either metals or explosives were found; however, the locations where the higher metal concentrations were found were not the locations with the highest concentrations of organic substances that could be related to ammunition. No indications were found that the observed concentrations of metals or organic substances originating from ammunition items were higher than during measurements in 1999, 2001, and 2003, even though degradation of the ammunition is expected to have progressed during the last 20 years. 

At the dump site, water samples were collected at ca. 20 cm above the seabed. The concentrations of explosives and their decomposition products were higher at some locations compared to other locations and also compared to reference samples taken outside of the dump site. For 2,4,6-TNT, local concentrations up to 57 ng/L water were measured. 

### 4.2. Comparison to Other Dump Sites around the World

Many countries, including the United States, United Kingdom, Canada, Japan, Russia, Germany, France, Italy, and others, manufactured large quantities of ammunition filled with nitroaromatic explosives for use in the two World Wars (WW I and WW II) [19]. Especially after World War II, the United States, France, the Soviet Union, and the United Kingdom decided on sea dumping of confiscated German munitions along, as well as off, the coastlines of the North and Baltic Seas. Until the 1970s, significant amounts of munitions and explosives were disposed in marine areas or in large lakes because that was considered safe and inexpensive [20].

As a result, 1.6 million metric tons of munitions was estimated to be dumped in the German parts of the North Sea, as well as 300,000 tons in the Baltic Sea [21]. In addition, sunken war ships armed with ammunition or wrecks with munitions cargo onboard are also a major source of munitions compounds in oceanic environments [19], as around 10,000 shipwrecks are present in the North Sea region alone, some 500 of them loaded with ammunition [22].

Numerous studies have measured munitions compounds like 2,4,6-TNT, 1,3,5-trinitro-1,3,5-triazinane (RDX), octahydro-1,3,5,7-tetranitro-1,3,5,7-tetrazocine (HMX), and others in seawater, sediments, and marine organisms (reviewed in [17,22,23,24,25,26]). Rosen et al. [27] investigated underwater concentrations of energetics such as TNT and RDX at the former Vieques Naval Training Range at Bahia Salina del Sur (Vieques, Puerto Rico, USA), a bay with documented high incidence of munitions. While, with passive sampling systems, TNT and RDX were observed at ultra-trace concentrations, in sediment and porewater samples munitions constituents were below detection limits (approximately 5 µg/kg and 5 ng/L, respectively). The authors conclude that despite the relatively high density of munitions at Bahia Salina del Sur, only low-level contamination exists on a very localized scale, suggesting that the sediment was not a sink for these constituents at this location. 

In recent approaches, novel and highly-sensitive analytical methods have been developed to allow detection of munitions compounds in the ng per liter or ng per kg range in sea water and sediment, respectively, and in the pg per g range in marine biota [11,17,22,28,29,30]. Dissolved munitions compounds were detectable in nearly all water column samples collected throughout the German Baltic Sea during the UDEMM project [28]. The reported concentrations had a range of nearly four orders of magnitude [17,28], most likely caused by rapid mixing and dilution away from solid explosive sources on the seafloor. 2,4,6-TNT concentrations of 1–10 ng per liter were found in most samples, which shows that the solubility limit was unlikely a major factor contributing to the low concentrations at the Dutch site. The low concentrations are more likely to be related to the combination of a low dissolution and release rate and fast refresh rate of the water flow due to the tidal current.

### 4.3. Munitions Compounds in Biota

Recently, Beck et al. [31] provided a comprehensive study on the accumulation of munitions compounds in field-collected biota. In the southwest Baltic Sea, ammunition-related compounds were detected in more than 98% of organisms collected (median ~1 ng/g). The Baltic Sea has an especially high presence of unexploded ordnance and dumped munitions, thereby representing a large potential source of toxic chemicals originating from explosives. In their study, plankton, macroalgae, tunicates, sponges, mollusks, echinoderms, polychaetes, anemones, crustaceans, and fish were analyzed for a variety of explosives and their derivatives including 2,4,6-TNT, 2-ADNT, 4-ADNT, 1,3-DNB, and RDX. 2,4,6-TNT was rarely detected in fish, while the transformation products ADNT and DANT were observed mainly in fish viscera rather than filet. In most cases, concentrations of 2,4,6-TNT and its transformation products ADNT and DANT were significantly higher in biota from the munitions dump site compared with other locations.

Strehse et al. [29] performed biomonitoring studies with blue mussels deployed at the sediment surface near scattered munitions in the Kolberger Heide in the southwest Baltic Sea. They found concentrations of 2,4,6-TNT, 2-ADNT, and 4-ADNT of 31, 104, and 131 ng/g, respectively. For mussels positioned 1 m above the sediment surface neither 2,4,6-TNT nor 2-ADNT could be detected, but 4-ADNT was detected at 9 ng/g wet weight [29]. 4-ADNT was detected at up to 8 ng/g (wet weight) in mussels deployed directly at moored mines [32]. Mussel monitoring studies at Kolberger Heide showed that blast-in-place operations, typically leaving substantial amounts of unreacted explosive material on the seafloor, may lead to considerably higher accumulations of 2,4,6-TNT and its transformation compounds in the exposed mussels. These concentrations are higher than the concentrations that could be detected in the Eastern Scheldt dump site, where active biomonitoring was performed in 2001 using baskets with mussels. After six weeks, the concentrations of 2,4,6-TNT, 2,4-DNT, 2-ADNT, and 4-ADNT in the mussel tissue were below the detection limit of 1 ng/g, and based on this, the detected amounts were concluded to be insufficient to pose human health issues, even after daily consumption of these mussels [7].

### 4.4. Toxicity and Biological Effects

Acute toxicity studies in laboratory aquariums routinely involved significantly higher exposure concentrations than those expected in the environment [16,18,23,33,34,35]. For example, the marine flatworm *Macrostomum lignano* was shown to stop feeding and starve when exposed to 2,4,6-TNT at 3000 ng/L, 2-ADNT at 330 ng/L, and 4-ADNT at 33 ng/L [36]. Koske et al. [37] recently proved genotoxicity in zebrafish embryos (*Danio rerio*) of 2,4,6-TNT and its metabolites 2-ADNT and 4-ADNT. Lethal concentrations (LC50) were 4500 ng/L for 2,4,6-TNT, 13,400 ng/L for 2-ADNT, and 14,400 ng/L for 4-ADNT. Competitive inhibition of 7-ethoxyresorufin-*O*-deethylase and 7-methoxyresorufin-*O*-deethylase by 2,4,6-TNT in vitro was demonstrated for three flatfish species sampled from the Baltic Sea [38]. Mariussen et al. [39] investigated the effects of 2,4,6-TNT in juvenile Atlantic salmon (*Salmo salar*). Mortality and severe hemorrhages in the dorsal muscle tissue near the spine, as well as effects on blood parameters such as glucose, urea, hematocrit, and hemoglobin, occurred in fish exposed to 1000 ng/L. The highest concentrations measured during the current research at the Eastern Scheldt dump site are at least two orders of magnitude lower than the concentrations reported above for toxicity, which makes it unlikely that biota in this region may be acutely affected. 

An interesting observation was made by Strehse et al. [40] with regard to chronic toxicity of 2,4,6-TNT in the blue mussel. While it was known that the mechanism of toxicity and carcinogenicity of 2,4,6-TNT and its derivatives occurs through its capability of inducing oxidative stress in the target biota, they found that 2,4,6-TNT can induce the gene expression of carbonyl reductase in blue mussels. Carbonyl reductases are members of the short-chain dehydrogenase/reductase (SDR) superfamily and, amongst others, metabolize xenobiotics bearing carbonyl functions, such as lipid peroxidation derived reactive carbonyls. Hence, carbonyl reductase provides a defense mechanism against oxidative stress and reactive oxygen species (ROS). In their study, they identified and cloned the gene coding for carbonyl reductase from the blue mussel *Mytilus* spp. by a bioinformatics approach. In both laboratory and free field studies, they could show that 2,4,6-TNT induces a strong and concentration-dependent induction of the gene expression of carbonyl reductase in the blue mussel. Carbonyl reductase may thus serve as a biomarker for 2,4,6-TNT exposure on a molecular level, which is useful to detect 2,4,6-TNT contaminations in the environment and to perform a risk assessment both for the ecosphere and the human seafood consumer [40]. Nevertheless, despite increasing research, our knowledge about the long-term effects of munitions compounds on marine species and ecosystems is still limited and more research is needed.

To the best of our knowledge, no directly applicable regulations exist at a global level with regard to authoritative regulations for the maximal tolerable concentrations of explosives and their decomposition products in surface waters. For drinking water, a 2 µg/L health advisory guideline for 2,4,6-TNT in drinking water was reported for lifetime intake, as well as a 100 µg/L health advisory level for a cancer risk of 10^−4^ [41]. The Eastern Scheldt water is not used for drinking water production, however, the highest concentrations measured in the water samples are a factor of 20 to 2000 lower than these guidelines. An ad hoc maximum permissible risk level of 0.62 µg/L was determined and reported for 2,4,6-TNT in ground water by Lijzen et al. [42]. The highest concentrations determined in the Eastern Scheldt were a factor of 10 lower than this level. 

## 5. Conclusions

The present study is part of the continuous monitoring of the Eastern Scheldt munitions dump site. The surfaced ammunition that was dumped in the Eastern Scheldt in the Netherlands after World War II was found to be severely degraded and leak paths through the casings were observed, making the explosives in the ammunition accessible to sea water. With new methods for analyzing nitroaromatic explosives that were recently developed at Kiel University, it was now possible, for the first time, to quantify explosive chemicals in this area in water and sediment. In the direct vicinity of ammunition, elevated concentrations of ammunition-related compounds (both metals and organic substances) were found. However, even though the water and sediment samples were collected as close to the ammunition items as possible, the concentrations of these compounds were low and, as far as available, no quality standards or limits were exceeded. The highest concentrations measured during the current research at the Eastern Scheldt dump site were at least two orders of magnitude lower than the concentrations reported for toxicity. In addition, during previous research, the concentrations of 2,4,6-TNT and its decomposition products in caged mussels were found to be at least an order of magnitude lower than in mussels from other dump sites. The presence of fouling, the low solubility of energetic compounds, and dilution by the high local water current were concluded to be the main causes for the absence of high concentrations of ammunition-related compounds. Nevertheless, the Eastern Scheldt dump site should routinely be monitored in the future.

## Figures and Tables

**Table 1:** GC-MS parameters of the different measurement methods.

	Explosives and Diphenylamine	Hexachloroethane	White Phosphorus
Inlet liner	Quartz wool	Quartz wool	SPME
Injection type	Liquid, 1 µL	Liquid, 1 µL	SPME, gray PDMS fiber
Injection temp.	260 °C	140 °C	290 °C
Column flow	1.5 mL × min^−1^	1.5 mL × min^−1^	1.2 mL × min^−1^
Oven temp.	100 °C (1 min) 30 °C/min to 220 °C (2 min), 70 °C/min to 280 °C (3 min)	40 °C (0.50 min), 30 °C/min to 150 °C, 80 °C/min to 280 °C (1 min)	50 °C (1 min), 25 °C/min to 300 °C (5 min)
Total run time	10.86 min	6.79 min	16.00 min
Transferline temp.	250 °C	250 °C	280 °C
Ion source temp.	300 °C	300 °C	300 °C
Ionization method	EI	EI	EI

**Table 2:** Retention times and quantification peaks of the different compounds.

Substance	Rt [min]	Precursor [m/z]	Quan. Peak [m/z]
1,3-Dinitrobenzene	4.68	122.0	75.0
2,4-Dinitrotoluene	5.05	165.0	119.1
Diphenylamine	5.43	169.1	168.2
2,4,6-Trinitrotoluene	5.80	210.0	164.1
^13^C^15^N-Trinitrotoluene	5.80	220.1	173.1
4-Amino-2,6-dinitrotoluene	7.27	197.0	180.1
2-Amino-4,6-dinitrotoluene	7.57	197.0	180.1
Hexachloroethane	4.29	200.9	165.9
White Phosphorus	4.91	-	123.9 *

* SIM [m/z] for quantitation.

**Table 3:** Concentrations of organic substances detected in water samples in nanograms per liter water. LoD and LoQ indicate limits of detection and limits of quantification, respectively. Indicated concentrations were measured after sample collection in 2020 (locations L1–L15), in 1999, and in 2001. At location L13, the water sample was collected at the surface.

	2,4,6-TNT	2,4-DNT	2-ADNT	4-ADNT	1,3-DNB	DPA	HCE
LoD	3	0.05	0.06	0.06	0.20	5	0.6 × 10^−3^
LoQ	3	0.15	0.20	0.19	0.66	5	2 × 10^−3^
L2	<LoD	<LoD	0.9	1.3	0.2	<LoD	14 × 10^−3^
L3	<LoD	1.1	0.9	1.3	0.6	<LoD	14 × 10^−3^
L4	5.5	0.6	1.4	1.9	1.1	<LoD	9 × 10^−3^
L6	44.5	1.1	11.4	10.0	1.0	<LoD	11 × 10^−3^
L7	56.7	0.8	13.0	11.2	1.1	<LoD	17 × 10^−3^
L10	15.1	0.4	4.6	4.2	1.1	<LoD	4 × 10^−3^
L11	<LoD	0.5	2.5	2.9	0.9	<LoD	18 × 10^−3^
L12	29.1	9.0	3.5	4.5	7.8	<LoD	17 × 10^−3^
L14	11.1	0.3	1.7	2.0	0.3	15	14 × 10^−3^
L15	13.6	<LoD	2.1	2.0	0.2	<LoD	24 × 10^−3^
L13	<LoD	0.8	1.1	1.3	0.2	<LoD	17 × 10^−3^
1999	<250	<250	<2000	<2000	n/a	n/a	n/a
2001	<10–500	<10–100	<10–60	<10–90	n/a	n/a	<100

**Table 4:** Concentrations of organic substances detected in sediment samples in nanograms per gram of dry sample. LoD and LoQ indicate limits of detection and limits of quantification, respectively. Indicated concentrations were measured after sample collection in 2020 (locations L1–L15), in 1999, in 2001, and in 2003.

	2,4,6-TNT	2,4-DNT	2-ADNT	4-ADNT	1,3-DNB	DPA	HCE
LoD	0.05	0.02	0.03	0.03	0.10	0.002	0.1 × 10^−3^
LoQ	0.15	0.08	0.10	0.10	0.33	0.007	0.1 × 10^−3^
L1	<LoD	<LoD	<LoD	<LoQ	<LoD	1.7	0.3 × 10^−3^
L2	<LoD	<LoD	<LoD	0.9	<LoD	1.1	0.2 × 10^−3^
L3	<LoD	<LoD	<LoD	<LoQ	<LoD	2.0	0.7 × 10^−3^
L4	<LoD	<LoD	<LoD	<LoQ	<LoD	2.0	0.2 × 10^−3^
L6	1.2	1.9	0.8	1.4	<LoD	0.9	0.7 × 10^−3^
L7	<LoD	<LoD	<LoQ	<LoQ	<LoD	1.5	0.5 × 10^−3^
L10	<LoD	<LoD	<LoQ	<LoQ	<LoD	2.6	0.3 × 10^−3^
L11	0.7	<LoD	0.5	<LoQ	<LoD	3.1	0.5 × 10^−3^
L12	<LoD	<LoD	<LoQ	<LoQ	<LoD	1.5	<LoD
L14	<LoD	<LoD	<LoQ	<LoQ	<LoD	1.7	0.2 × 10^−3^
L15	<LoD	<LoD	<LoQ	<LoQ	<LoD	6.9	0.3 × 10^−3^
1999	<10	<10–3000	<100	<100	n/a	n/a	n/a
2001	<1	<1	<1	<1	n/a	<1	<1
2003	<0.1–5.9	n/a	<0.1–11.3	<0.1–8.9	n/a	n/a	n/a

**Table 5:** Concentrations of metal detected in water samples in micrograms per liter water. Indicated concentrations were measured after sample collection in 2020 (locations L1–L15), in 1999, and in 2001. At location L13, the water sample was collected at the water surface.

	Fe	Cu	Ni	Zn	Sn	Mo	Co	Pb	Cd
L1	0.37	4.1	0.9	7.4	0.81	10.1	0.04	0.09	0.04
L2	0.43	4.4	1.4	19.2	0.08	9.6	0.06	0.10	0.05
L3	0.42	4.3	1.1	13.3	0.04	9.5	0.04	0.04	0.05
L4	0.39	11.9	2.4	74.7	0.03	10.0	0.05	0.05	0.38
L6	0.37	5.0	1.2	23.1	0.02	9.6	0.03	0.04	0.05
L7	0.37	3.5	1.2	19.5	0.02	9.6	0.03	0.03	0.03
L10	0.40	13.2	2.5	34.6	0.04	9.9	0.06	0.05	0.09
L11	0.41	11.3	1.5	75.5	0.02	9.3	0.05	0.38	0.04
L12	0.38	2.8	1.0	22.6	0.01	9.3	0.03	0.02	0.05
L14	0.34	7.6	1.5	49.6	0.02	9.1	0.03	0.36	0.06
L15	0.36	3.1	0.9	11.7	0.01	9.2	0.03	0.02	0.02
L13	0.41	0.9	0.6	1.6	0.01	9.1	0.04	0.01	0.01
1999	n/a	0.3–21.4	0.6–8.3	2.0–26.6	0.0–2.0	6.7–9.4	0.0–0.1	0.1–0.8	0.1
2001	n/a	0.7–5.1	0.3–0.8	6.4–48	0.1–1.0	5.1–8.5	0.4–0.6	1.4–4.0	0.0–0.1

**Table 6:** Concentrations of metal detected in sediment samples in nanograms per gram of dry sample. Indicated concentrations were measured after sample collection in 2020 (locations L1–L15), in 1999, and in 2001.

	Fe	Cu	Ni	Cr	Zn	Sn	Mo	Co	Pb	Cd
L1	9.1 × 10^3^	4.9	6.8	27	33	1.4	1.1	3.2	12.5	0.17
L2	5.6 × 10^3^	3.2	4.5	20	23	0.9	0.7	2.2	8.7	0.12
L3	7.7 × 10^3^	4.3	6.1	28	30	1.2	0.6	2.8	10.6	0.12
L4	10.0 × 10^3^	5.2	5.0	20	26	1.3	0.5	2.5	9.3	0.07
L6	16.0 × 10^3^	7.3	4.1	13	82	1.8	0.6	3.2	10.9	0.03
L7	9.9 × 10^3^	3.7	3.9	12	215	0.9	0.5	2.3	7.1	0.05
L10	7.9 × 10^3^	71.6	5.0	22	33	1.9	0.4	2.5	12.2	0.11
L11	9.7 × 10^3^	9.5	6.6	25	40	3.7	0.5	3.1	15.6	0.25
L12	20.6 × 10^3^	5.0	4.7	19	33	1.3	1.4	2.3	12.7	0.11
L14	5.3 × 10^3^	19.9	2.5	9	25	1.6	0.2	1.7	14.6	0.03
L15	6.0 × 10^3^	18.9	4.1	16	51	0.8	0.3	2.1	10.7	0.06
1999	0.7 × 10^3^–1.07 × 10^3^	n/a	<10	n/a	<10–37	n/a	n/a	<10	<10–29	n/a
2001	2.8 × 10^3^–21 × 10^3^	<25	<25	<50	<25–225	<25	<25	n/a	<25	n/a

## Data Availability

The data presented in this study are available in Den Otter, A.; Olde, M.; Van der Heijden, A.; Koolloos, M. Monitoring munitiestort Oosterschelde 2020.
